# Retraction: Synthesis of xanthone derivatives and anti-hepatocellular carcinoma potency evaluation: induced apoptosis

**DOI:** 10.1039/d2ra90041f

**Published:** 2022-04-27

**Authors:** Laura Fisher

**Affiliations:** Royal Society of Chemistry Thomas Graham House, Science Park, Milton Road Cambridge CB4 0WF UK advances-rsc@rsc.org

## Abstract

Retraction of ‘Synthesis of xanthone derivatives and anti-hepatocellular carcinoma potency evaluation: induced apoptosis’ by Jie Liu *et al.*, *RSC Adv.*, 2019, **9**, 40781–40791, https://doi.org/10.1039/C9RA06408G.

The Royal Society of Chemistry hereby wholly retracts this *RSC Advances* article. There is a significant amount of unattributed text overlap with articles by different author groups that were not cited, including articles published in *Scientific Reports* by Zhenjian Zhuo *et al.*^1^ and *Carcinogenesis* by Dong-Mei Zhang *et al.*^2^

In addition, the raw data provided by the authors could not be used as verifiable raw data to validate the published western blot images.

The considerable amount of unattributed text overlap, and lack of verifiable raw data, undermines the integrity and reliability of the entire article.

Jie Liu agrees to the retraction. The other authors have been informed but have not responded to any correspondence regarding the retraction.

Signed: Laura Fisher, Executive Editor, *RSC Advances*

Date: 29th March 2022

## Supplementary Material
